# The Effect of Intradialytic Exercise Using Virtual Reality on the Body Composition of Patients with Chronic Kidney Disease

**DOI:** 10.3390/nu16121968

**Published:** 2024-06-20

**Authors:** Marta Romeu-Perales, Eva Segura-Ortí, Alicia Cana-Poyatos, Marina Toquero-Correa, Vicent Benavent-Caballer, Delia Pans-Alcaina, Rafael García-Maset, Alicia García-Testal

**Affiliations:** 1 Department of Biotechnology, Universidad de Valencia, 46010 Valencia, Spain; martaromeu2001@gmail.com; 2 Department of Physiotherapy, Universidad Cardenal Herrera-CEU, CEU Universities, 46115 Valencia, Spain; marinatc112@gmail.com (M.T.-C.);; 3 Research Unit, Nephrology Service, Hospital de Manises, 46940 Valencia, Spain; cana_ali@gva.es; 4 Hemodialysis Unit Nursing, Nephrology Service, Hospital de Manises, 46940 Valencia, Spain; 5 Nephrology Service, Hospital de Manises, 46940 Valencia, Spainagtestal@gmail.com (A.G.-T.)

**Keywords:** exercise, chronic kidney disease, hemodialysis, virtual reality

## Abstract

Background: Individuals with chronic kidney disease (CKD) often experience reduced muscle strength and diminished health-related quality of life (HRQoL), and engaging in regular exercise may improve them. The aim of this study was to assess the effect of intradialytic exercise using non-immersive virtual reality (VR) on body composition of patients with CKD on hemodialysis (HD). Methods: This was a substudy in a clinical trial of intradialytic exercise intervention using a non-immersive VR game in which the patient interacted by moving the lower limbs. Body composition was determined by BCM Fresenius multifrequency stereoscopic bioimpedance. Body mass index (BMI), fat tissue index (FTI), lean tissue index (LTI), extracellular/intracellular water (EIW), and phase angle (PA) were recorded in 52 patients, 24 in the control group (CG) and 28 in the exercise group (EG). Results: Statistically significant differences were observed between both groups. The LTI increased in the EG while it decreased in the CG. The FTI and the EIW decreased in the EG compared to the increase observed in the CG. Conclusions: Intradialytic exercise using non-immersive VR was associated with an increase in LTI and a decrease in FTI of CKD patients on HD.

## 1. Introduction

CKD (CKD) stage 5 is a pathology associated with high morbidity and mortality, mainly of cardiovascular causes. It is, therefore, an important public health burden. Hemodialysis (HD) is the most common form of renal replacement therapy [1,2].

Exercise is an intervention that results in important benefits for people undertaking HD [3,4,5,6], such as improved functional capacity, physical function, muscle strength, mood, HRQoL, and survival [4,5]. In addition, aerobic exercise and muscular endurance during HD improves blood pressure control, lipid profile, and mental health in this population [3,4,5,6]. Intradialytic exercise has been recommended due to its high tolerance and its ability to promote adherence. Despite the benefits of exercise during dialysis, it is not routinely implemented in clinical practice. The literature reports barriers identified in patients, such as muscle fatigue, pain, lack of time, lack of an exercise partner, or lack of place to exercise [7]. Barriers from health care professionals include the lack of experience and time [8]. The inclusion of exercise experts into the health care programs for HD patients could provide a deeper understanding of the barriers and benefits of exercise [7]. As an alternative to overcome these barriers, a new method of intradialytic exercise based on non-immersive virtual reality (VR) video games has recently been developed [9,10]. It has been shown in a pilot study that VR exercise was feasible to perform in HD with no differences in results or adherence compared to a combined exercise program [9]. These results were confirmed in another study with a 12-week program that demonstrated significant results on physical function in a broad battery of functional tests [11]. Furthermore, intradialysis exercise using VR was safe without presenting hemodynamic instability [10,12], as well as possible benefits in the reduction in the consumption of healthcare resources and health micro-costing [13]. This type of program is designed to be like a game, which can help patients perceive it as enjoyable, and is also easier for dialysis staff to implement, compared to the conventional exercise training sessions.

Throughout life, body composition undergoes a variety of changes influenced by genetic factors, hormonal shifts, and lifestyle. Among the observed changes, variations in body mass, fat, muscle, and bone tissue are remarkable. Increased fat mass and decreased muscle and bone mass can be associated with several pathologies [14]. The assessment of body composition is important both for the clinical and the research field. Body composition is related to health status and to the risk of chronic degenerative disease, and in addition, it is employed to accurately assess nutritional status [15].

One of the methods for assessing body composition is bioelectrical impedance (BIE). BIE is an inexpensive, easy, portable, and reproducible method. The “European Working Group on Sarcopenia in Older People” recommends BIE for monitoring muscle mass in the assessment of sarcopenia [16]. The technique is based on the principle of the conductivity of water and the resistance to the passage of an alternating electric current in the body. Single-frequency and multifrequency BIE are available [15,17]. Multifrequency BIE allows the assessment of the patient’s nutritional and hydration status [17].

CKD has an impact on the body composition of patients [18]. CKD patients have a high prevalence of sarcopenia due to increased muscle protein catabolism [19] and, consequently, low functional capacity, disability, frailty, decreased HRQoL levels, and increased mortality [20,21]. After learning about the importance of body composition as a risk factor for morbidity and mortality, interest in measuring it in CKD has increased [17]. Bioimpedance has been used in these patients for monitoring parameters such as muscle mass, fat content, or hydration [22].

Previous studies have reported the effect of exercise on muscle mass and body composition in CKD patients on HD, and their results have been conflicting [22,23,24,25,26]. Several studies [24,25] reported a significant increase in lean mass after exercise training; however, other studies reported a decreased LTI [22]. A systematic review including 14 randomized clinical trials analyzed the impact of physical activity on body composition in CKD stage 5 undergoing HD [26]. The results regarding percentage of body fat, lean body mass, and BMI were not clear; it is to be noted that the existing evidence is insufficient to prove significant beneficial effects of exercise training on body composition. Regarding VR exercise, one study implemented an exercise program before the HD session and showed a significant increase in skeletal muscle mass in the exercise group [23]. To our knowledge, no previous study has reported the results of an intradialytic VR exercise on the body composition of patients undertaking hemodialysis.

The hypothesis of the present study was that an intradialytic exercise intervention using non-immersive VR would change the body composition of CKD patients on HD. Therefore, the aim of this study was to assess the effect of intradialytic exercise using non-immersive RV on the body composition of patients with CKD on chronic HD treatment.

## 2. Materials and Methods

### 2.1. Design

This was a quasi-experimental before–after substudy, in a clinical trial with open recruitment for 2 years. The clinical trial consisted of an intradialytic exercise intervention using the non-immersive VR program for 12 months.

The inclusion criteria were being on HD treatment for at least 3 months and being clinically stable. The exclusion criteria were presenting with a myocardial infarction in the 6 weeks prior to the intervention, unstable angina, amputation of both lower limbs above the knee without a prosthesis, cerebrovascular disease in the last 6 months such as stroke or transient ischemia or with relevant sequelae in the mobility of the lower limbs, and musculoskeletal or respiratory disorders that worsened on exertion.

All patients participating in the clinical trial who met the following criteria were included in this substudy. The control group (CG) included patients who participated in the clinical trial for less than 3 months and dropped out voluntarily, and, at the time of the present study, have not been engaging in exercise for more than 6 months. Patients who dropped out due to an incident pathology were excluded from the control group. The exercise group (EG) included patients who participated in the clinical trial for at least 9 months.

This study was registered at Clinical Trials (NCT04046042). This study obtained ethical approval (approval number 2018/0833) by the *Comité de Ética de la Investigación con medicamentos; Hospital Universitario y Politécnico La Fe.*

### 2.2. Intervention

The VR program consisted of a video game administered using a computer, a screen, and a motion-sensing camera. “Treasure Hunt” is a non-immersive VR game in which the patient must catch targets and avoid obstacles by moving the lower limbs. Participants started with short gaming times (1 set of 3 min) and progressed to the longest possible gaming time (6 sets of 6 min, with 1 min rest between sets). The gaming time progressed depending on the perceived exertion scale (exertion between 6 and 20, with 6 being minimal effort and 20 being maximum effort). If the participant perceived the session as 11 or lower, the exercise time increased. For further information about exercise load progression, please see these references [9,11].

The patients carried out individually adapted exercise sessions, depending on the RPE. Data on RPE were recorded in every exercise session, and the aim was that the participant perceived the session between 12 and 15 out of 20. If the participant felt the session as 11 out of 20 for three consecutive sessions, gaming time progressed.

The participants performed 5 min of warming-up and cooling-down exercises at the start and end of the program, respectively.

Exercise was offered in all HD sessions (3 times a week) to all the included patients.

### 2.3. Measurements

Body composition was determined by BCM Fresenius^®^ (Bad Homburg v. d. Höhe, Germany) multifrequency stereoscopic bioimpedance, based on the resistance or opposition to the passage of an applied electrical current. This is a BIE multifrequency that determines bioimpedance using values from 3 to 1000 kHz [27].

Body composition measurements were taken using BCM before the dialysis session at two moments: baseline (before starting the exercise program) and final, between 9 and 12 months after the start of the program. The following was recorded at each measurement: body mass index (BMI) kg/m^2^, fat tissue index (FTI) kg/m^2^, lean tissue index (LTI) kg/m^2^, extracellular/intracellular water (EIW), and phase angle (PA) degree.

Variables on metabolic–nutritional control were recorded, such as albumin (g/dL), hemoglobin (g/dL), potassium (mmol/L), phosphorus (mg/dL), calcium (mg/dL), ferritin (ng/mL), bicarbonate (mmol/L), triglycerides (mg/dL), and LDL and HDL cholesterol (mg/dL) determined in peripheral blood.

The descriptive variables included age (years), sex (female/male), etiology of kidney disease, history of diabetes mellitus (yes/no), time on hemodialysis treatment (years), vascular access (central venous catheter CVC/arteriovenous fistula AVF), and hemodialysis technique (low-flow hemodialysis, high-flow hemodialysis, online hemodiafiltration), Charlson index (CI), and dialysis dose in monocompartimental Kt/V, determined in peripheral blood. The electronic/digital medical record of each patient was the data source.

### 2.4. Statistical Analysis

The sample size was estimated using the GRANMO sample size calculator, Version 7.12 April 2012 of the Program of Research in Inflammatory and Cardiovascular Disorders, Institut Municipal d’Investigació Mèdica, Barcelona, Spain: https://www.imim.es/ofertadeserveis/software-public/granmo/ (accessed on 15 January 2019). As reference values, we used the results of previous studies [24,25]. So, accepting an alpha risk of 0.05 and a beta risk of 0.2 in a two-sided test, 36 subjects were necessary in the first group and 36 in the second to identify a statistically significant difference greater than or equal to 1 unit for lean tissue index. The common standard deviation was assumed to be 2 and the correlation coefficient between the initial and final measurement as 0.8. The drop out rate was estimated to be 30%.

Quantitative variables were described as mean (standard deviation) and Qualitative variables were described in absolute-number percentages (%). Regarding the statistical methods, in the first approach, a univariate analysis was carried out to compare the difference in the body composition of the participants before and after completing the study in both groups: exercise/control. For this, Student’s *t* test was used for paired and parametric data of continuous variables and the Mann–Whitney/Wilcoxon rank-sum test for non-parametric paired data.

In the second approach, a multiple linear regression analysis was carried out where the result variable was the difference between the values of body composition before and after this study, and the independent variables were the age, gender, and group (exercise/control) of all the participants. Probability *p* values less than 0.05 were considered statistically significant. All the analyses were performed using R packages available for this type of study, version 4.2.0 (R Foundation for Statistical Computing, Vienna, Austria).

## 3. Results

**Figure 1:**
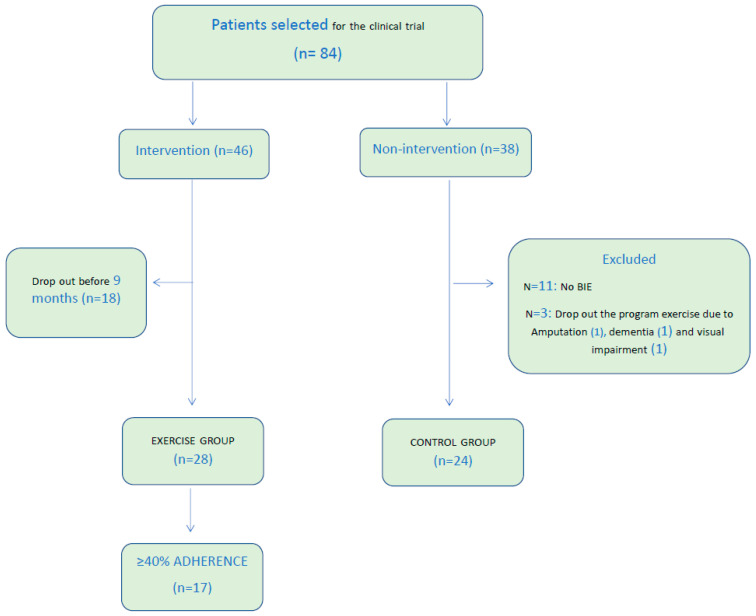
Flowchart of patients participating in the control group and exercise group in this study.

Eighty-four patients were included. After the losses reflected in the flowchart (Figure 1), 52 patients remained in this study, 24 were in the control group and 28 in the exercise group. The general median age of the participants was 73.5 years, with 35% being women. These baseline characteristics (quantitative and qualitative variables) are listed in Table 1.

Regarding adherence, the patients in the exercise group performed an average of 42% of the exercise sessions offered.

A first analysis of the changes in body composition of the final measurement, determined between 9 and 12 months after the start of the exercise program, compared to the baseline measurement, was carried out. The results of the comparison between groups are shown in Table 2.

Significant differences in FTI and EIW were observed in the whole exercise group (N = 28); however, the changes on LTI were significant only in patients who performed more than 40% of the exercise sessions offered (N = 17).

**Figure 2:**
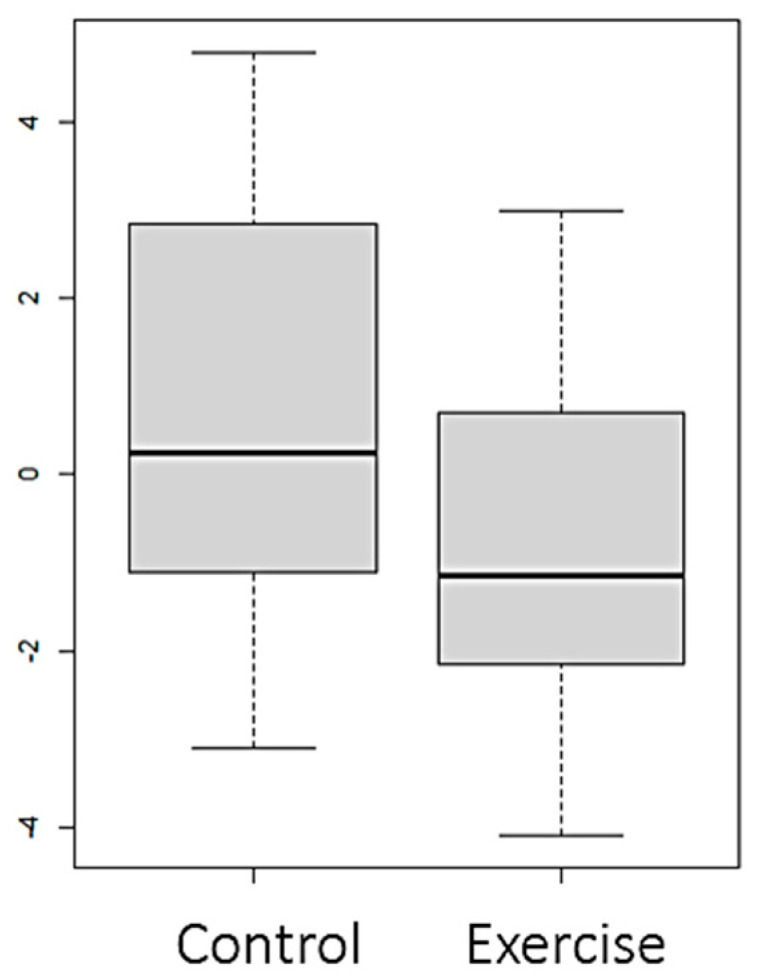
Changes (Final–Baseline) at the end of the intervention in fat tissue index (FTI) (kg/m^2^).

**Figure 3:**
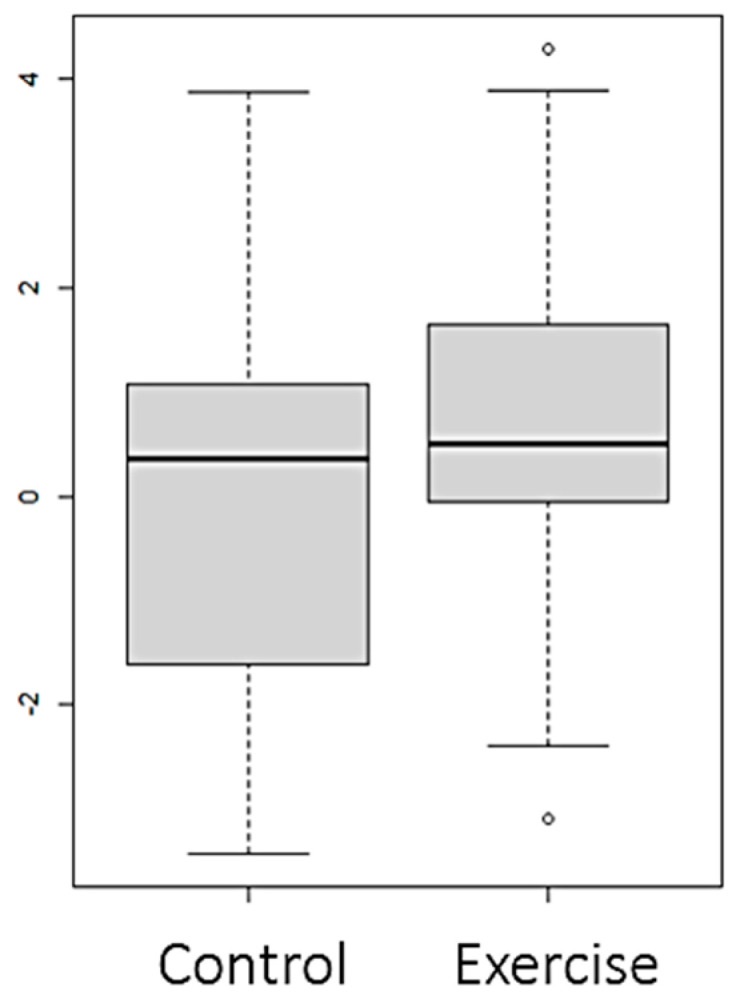
Changes (Final–Baseline) at the end of the intervention in lean tissue index in the whole sample (LTI) (kg/m^2^).

**Figure 4:**
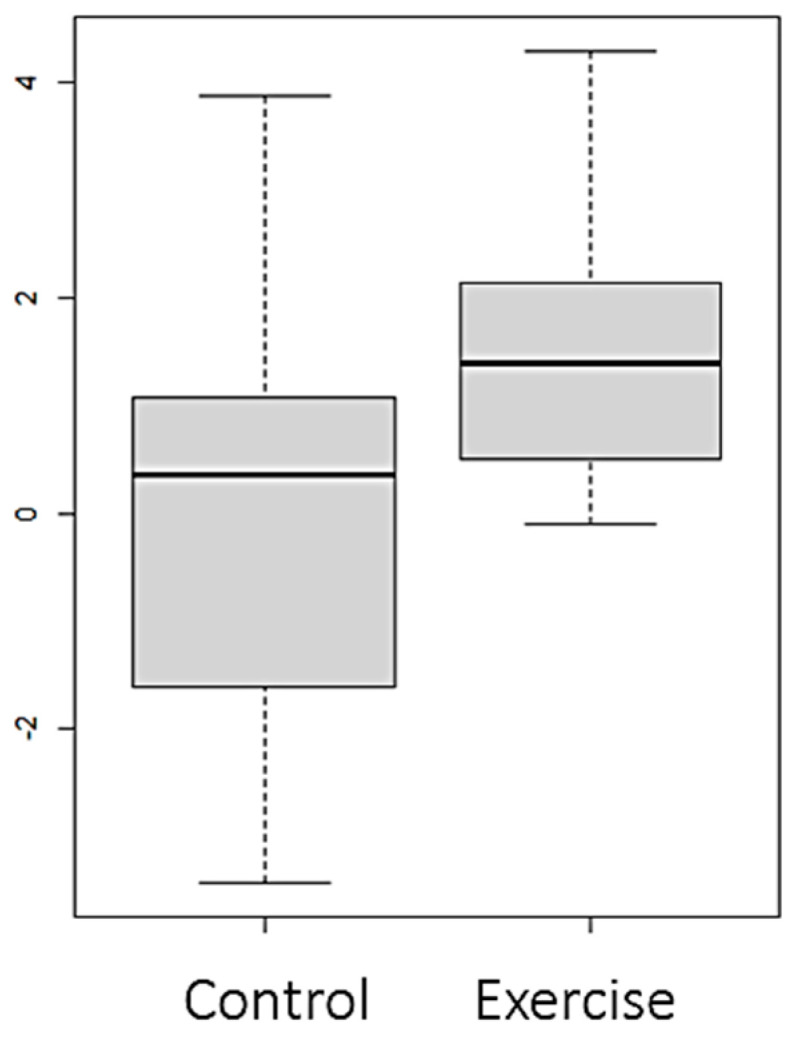
Changes (Final–Baseline) at the end of the intervention in lean tissue index (LTI) (kg/m^2^); patients in the exercise group with adherence ≥ 40% (N = 17).

**Figure 5:**
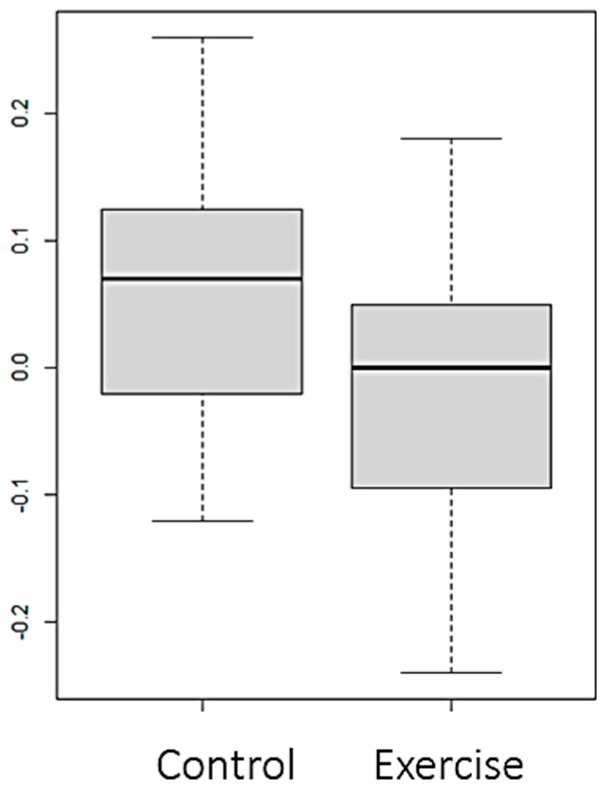
Changes (Final–Baseline) at the end of the intervention in extracellular/intracellular water (EIW).

**Figure 6:**
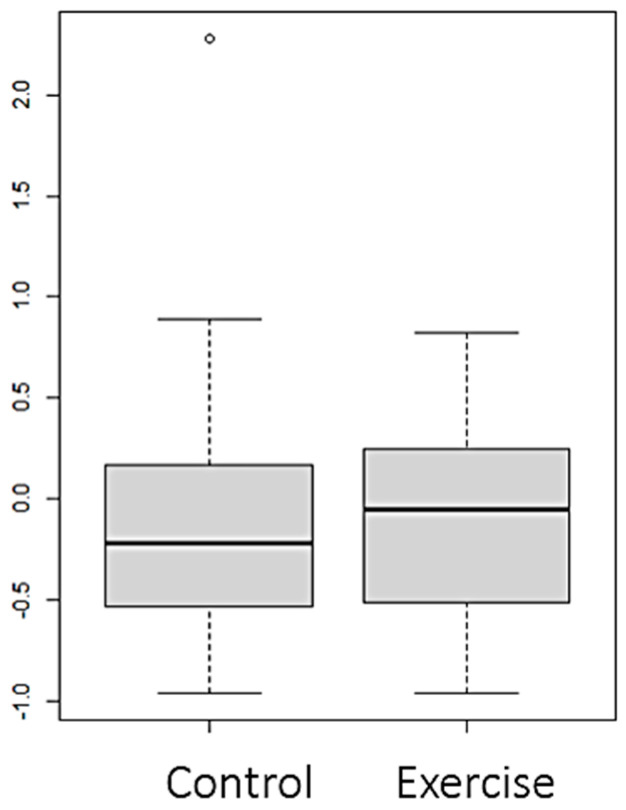
Changes (Final–Baseline) at the end of the intervention in phase angle degree (PA).

Statistically significant differences (*p* value < 0.05) were observed between both groups in the variables FTI (Figure 2), LTI (Figure 3 and Figure 4), and EIW (Figure 5). In the exercise group, LTI increased, while it decreased in the control group; FTI and EIW decreased in the exercise group compared to the increase observed in the control group. Changes in PA are shown in Figure 6.

Table 3 shows the regression analysis of the changes (final—baseline) in body composition of the exercise group compared to the control group for the variables with significant association in the initial analysis (FTI, LTI, EIW, PA). The effect was corrected for age and sex. For the full results, see Supplementary Material (Table S1).

Several metabolic–nutritional variables were controlled. Table 4 shows the results of the analysis using the Mann–Whitney U Test, final minus baseline, with no differences between groups.

## 4. Discussion

The present study shows an increase in lean mass and a decrease in fat mass in hemodialysis patients after an intradialytic exercise program using VR. These effects are relevant in two ways. On the one hand, improving lean mass can result in important benefits linked to the treatment of sarcopenia. On the other hand, these results reflect the positive effects produced by a VR exercise program designed to facilitate intradialytic exercise for CKD patients.

Sarcopenia in patients with CKD on HD is associated with decreased functional capacity, increased morbidity, increased mortality, and decreased HRQoL [20,28]. Intervention is recommended in sarcopenia through exercise programs and nutrition guidelines [29]. The importance of exercise training has been pointed out due to the positive changes it produces on patients’ muscles, both in terms of structure and function [30]. However, exercise during HD is not considered a clinical routine, and exercise adherence is poor [9]. The implementation of exercise programs is well below the needs. That is why new methods have been proposed to generalize the practice, such as intradialytic exercise or incorporating new technologies. Recently, the benefits in strength, functional capacity and HRQoL of exercise based on non-immersive VR videogames specifically designed to be performed during HD have been verified [10,11]. In previous studies of VR exercise during HD treatment, after 12 weeks of exercise, physical function improved, compared to the control group [11]. Until now, its effects on muscle mass were unknown.

In healthy adults, resistance training not only increases muscle strength and improves physical function but also increases skeletal muscle mass compared to the non-exercising controls. Load and weekly frequency impacted increases in muscular strength but not muscle hypertrophy. Volume (number of sets) influenced muscular strength and hypertrophy. Contraction type also impacted skeletal muscle hypertrophy (eccentric-favored) [30]. However, previous studies on the effects of exercise in HD patients have shown conflicting results [26].

A VR exercise program using Nintendo’s Wii Fit Plus (tool created for the general population) was implemented in HD patients prior to the dialysis session. This study showed improvements in skeletal muscle mass measured using an Inbody s10 body composition analyzer (Biospace, Seoul, South Korea), from 25.1 kg to 26.2 kg, while there were no significant differences in the control group. In addition, the exercise group showed a decrease in body fat of 0.8% [23]. The present study used a platform developed specifically to implement an intradialytic exercise program for patients on HD.

In the study by Kopple et al. [31], resistance and combined exercises were performed three times per week. Resistance training was performed immediately before the start of HD treatment, and aerobic training was performed during the first 60 min of each HD session. The exercise program lasted 18 weeks. They observed an increase in lean body mass of 0.4 kg in the case of resistance exercises and an increase in lean body mass of 0.5 kg in the case of combined exercises in the exercise group. In the non-active group, lean mass increased by 0.7 kg. Differences between groups were not significant [31]. Similar results were obtained in the study by Marinho et al. [32], noting a non-significant increase in lean mass in both groups. Chen et al. [33] who studied the effect of strength training during HD, observed that the exercise group increased, although not significantly, the value of lean body mass by 2.1 kg [33]. Lopes et al. [34] and Rosa et al. [35] observed the effect of resistance and strength intradialytic exercises, respectively, three times per week for 12 weeks. They did not find significant changes. In the subjects who did not exercise, it decreased or presented a smaller increase. There were no significant differences between groups [34,35].

On the contrary, in the study by Johansen et al. [36], 12 weeks of resistance exercise, performed during HD sessions three times a week, resulted in decreased lean body mass in both the exercise and control groups, with the decrease being greater in the exercise group with no significant difference between groups. In the exercise group, it decreased 0.4 kg, while in the control group, the value of lean body mass decreased 0.2 kg. Yuguero et al. [22] reported that an intradialytic exercise program performed twice a week for 6 months with high adherence (70.8%) was associated with a significant improvement in exercise capacity, strength, adherence, physical function, and HRQoL. Regarding the variation in body composition, they found a significant increase; the patients who exercised increased their body weight by almost 1 kg, with an increase in BMI and FTI (increase of 1 kg/m^2^). However, LTI decreased by 0.5 kg/m^2^, although this turned out not to be a statistically significant change [22].

Vasilki et al. [24] aimed to determine the effects of a 4-month intradialytic exercise program on functional capacity and body composition in patients who were candidates for kidney transplantation undergoing HD treatment and included 15 participants in the exercise group and 14 participants in the usual care group. As in our study, the analysis of body composition indices was performed using BIA. At the end of Vasilki et al.’s study, the exercise group had a significant increase in lean mass of 2.7%. In addition, there was a significant reduction of 6.6% in the fat mass index. These results report a trend of change in body composition similar to that observed by our team after an exercise program using VR. Torres et al. [25] observed a similar effect in HD patients after 3 months of exercise training. The final LTI was 16.2 ± 2.9 kg/m^2^, while the baseline value was 14.9 ± 3.7 kg/m^2^. In the present study, we have been able to verify improvements in lean mass and a decrease in fat tissue after applying an intradialytic exercise program through VR designed specifically for HD patients, reaching an increase in LTI in the exercise group of 1.5 kg/m^2^ for patients with adherence to exercise greater than 40% of the sessions. In addition, the FTI decreased by 0.75 kg/m^2^.

Therefore, the results in previous studies are contradictory, most frequently showing an increase in muscle mass, although not always significant. Longer interventions, such as the one developed in the present study, may be necessary to observe the results of exercise on the muscle mass of HD patients. Furthermore, the changes on LTI were significant only in patients who performed more than 40% of the offered exercise sessions. So, longer interventions with greater adherence could be the key to significant results on muscle mass in HD patients.

The main limitation of this study was the lack of randomization in the groups. This study was expressly designed in this way, since the benefits of exercise in HD patients are well known; so, not offering this intervention to some of the participants would not have been ethical. Thus, the exercise was offered to all patients who agreed to participate, and only those who participated for less than 3 months and had not performed the program for more than 6 months at the time of the measurements were included in the control group. We consider that, after this time, we can accept these patients as a control group. After overcoming the ethical limitations, it is important to consider that the patients who dropped out of the program may differ from those who continued. This limitation persists in our methodology. However, this was the best possible control group considering that having a non-exercise group was not deemed ethically acceptable. Previous studies have used patients who have not completed exercise programs as the control group [4], thus avoiding leaving patients out without offering intervention. Another limitation was that the determination of the lean mass of a patient on HD can be affected by the fluid balance [26]. We believe that this factor did not overestimate the lean mass results of the patients in the exercise group, because these patients decreased the ratio of extracellular/intracellular water after the exercise program. As described in the KDOQI 2020 nutrition guide “KDOQI Clinical Practice Guide-line for Nutrition in CKD: 2020 Update” [37], it is recommended that body composition is measured using BIE 30 min after the end of the HD session. However, the guidelines accept pre-dialysis measurements; conducting them after the session has ended presents challenges. In this sense, it is relevant to always perform the BIE at the same time for it to be comparable, and this was performed in the present study. On the other hand, this study did not control possible changes in the nutritional habits of the participants; the same dietary recommendations were made for the patients in the exercise group and the control group. These recommendations were the general recommendations for HD patients. Moreover, changes in basic nutritional variables were controlled without observing differences between the groups.

Regarding the VR exercise program applied in this study, we must specify that it was a low-intensity resistance training exercise program, since playing the VR translates into many repetitions of hip flexion and abduction with no added weight (only movement against gravity). Therefore, exercise of greater intensity (adding loads) could result in larger results. Another relevant limitation was adherence to the program, which could attenuate the effect. The results obtained in previous studies [9] report that adherence to the exercise program is more important than the type of exercise in achieving significant changes in the physical function of patients with CKD on HD. In our study, we have separately analyzed the effect on lean mass for patients with adherence greater than 40% of the sessions offered, evidencing positive effects. This measure can serve as the minimum adherence objective to be pursued in future programs, in the quest for effects on lean mass. Finally, we did not compare VR exercise with conventional intradialytic exercise regarding the impact on body composition. Future studies should clarify which is the best exercise modality.

## 5. Conclusions

In conclusion, intradialytic exercise using non-immersive VR is associated with an increase in lean tissue and a decrease in fat tissue in the body composition of CKD patients undergoing chronic HD treatment.

## Figures and Tables

**Table 1:** Descriptive variables of the control group and exercise group.

Variable	Control Group n = 24	Exercise Group n = 28
Age (years)	63.2 (19.8)	73.4 (10.7)
Gender (women)	9 (37.5)	9 (32.1)
Etiology of chronic kidney disease		
Renal vascular disease	3 (12.5)	5 (17.9)
Glomerulonephritis	3 (12.5)	2 (7.1)
Diabetic nephropathy	7 (29.2)	10 (35.7)
Unknown etiology	11 (45.8)	11 (39.3)
Mellitus diabetes (yes)	11 (45.8)	11 (39.3)
Charlson index	7.3 (3.1)	7.9 (2.5)
Time in hemodialysis (months)	48.7 (35.7)	50.5 (57.5)
Hemodialysis technique		
HFH	15 (62.5)	21 (75.0)
LFH	1 (4.2)	1 (3.6)
OLHDF	8 (33.3)	6 (21.4)
KtV	1.6 (0.2)	1.8 (0.5)
Body mass index (kg/m^2^)	25.9 (5.1)	28.2 (5.2)
Fat tissue index (kg/m^2^)	12.9 (5.8)	16.4 (6.5)
Lean tissue index (kg/m^2^)	12.0 (3.0)	16.2 (8.5)
Extracellular/intracellular water	1.0 (0.1)	1.1 (0.1)
Phase angle degree	4.1 (0.8)	4.0 (0.8)
Albumin (g/dL)	3.86 (0.35)	3.83 (0.38)
Hemoglobin (g/dL)	11.13 (1.30)	11.53 (1.15)
Potassium (mmol/L)	5.15 (1.40)	5.20 (0.66)
Phosphorus (mg/dL)	4.69 (1.54)	4.32 (1.29)
Calcium (mg/dL)	8.94 (0.66)	8.82 (0.44)
Ferritin (ng/mL)	513.74 (339.31)	565.17 (286.92)
Bicarbonate (mmol/L)	22.07 (3.62)	23.76 (3.09)
Triglycerides (mg/dL)	176.25 (108.83)	132.65 (58.19)
Cholesterol LDL (mg/dL)	66.10 (25.12)	55.51 (22.14)

Quantitative variables in mean (SD) and qualitative variables in absolute value (%) in the same column. HFH: high-flow hemodialysis; LFH: low-flow hemodialysis; OLHDF: online hemodialysis.

**Table 2:** Variation of body composition (Final–Baseline) in the control group and the exercise group.

Variable ^a^	Control Group	Exercise Group	*p* Value
BMI (kg/m^2^)	−0.097	−0.021	0.934
FTI (kg/m^2^)	0.725	−0.757	0.016 *
LTI ^b^ (kg/m^2^)	−0.383	1.506	0.001 *
EIW	0.047	−0.024	0.021 *
PA degree	−0.083	−0.106	0.614

Legend: BMI: Body mass index; EIW: Extracellular/intracellular water; FTI: Fat tissue index; LTI: Lean tissue index; PA: Phase angle. Statistical analysis on BMI and PA using the Mann–Whitney non-parametric test; FTI, LTI, and EIW were analyzed using Student’s *t* test. * *p* value < 0.05. ^a^ The results expressed in the table correspond to the change (Final–Baseline). ^b^ Patients in the exercise group with adherence to sessions ≥ 40% (N = 17).

**Table 3:** Regression analysis of changes in body composition in the exercise group compared to the control group.

Variable ^a^	Estimate	Std. Error	Lower 95%	Upper 95%	*p* Value
FTI (kg/m^2^)	−1.2	0.598	−2.402	0.002	0.05
LTI ^b^ (kg/m^2^)	2.110	0.551	0.993	3.228	0.001 *
EIW	−0.084	0.031	−0.015	−0.021	0.01 *
PA degree	0.087	0.163	−0.242	0.415	0.59

Legend: EIW: Extracellular/intracellular water; FTI: Fat tissue index; LTI: Lean tissue index; PA: Phase angle. Variables are studied by multiple linear regression analysis. * *p* value < 0.05. ^a^ The results expressed in the table correspond to the change (Final–Baseline). ^b^ Patients in the exercise group with adherence to sessions ≥ 40% (N = 17).

**Table 4:** Mann–Whitney U test analysis of changes in metabolic–nutritional variables in the exercise group compared to the control group.

	Exercise Group N = 28	Control Group N = 24	*p*-Value
Albumin (g/dL)	−0.16 (0.32)	−0.12 (0.42)	0.8153
Hemoglobin (g/dL)	−0.01 (1.14)	0.20 (1.87)	0.8502
Potassium (mmol/L)	−0.09 (0.72)	0.17 (0.84)	0.2092
Phosphorus (mg/dL)	−0.35 (1.37)	−0.44 (1.38)	0.8765
Calcium (mg/dL)	0.04 (0.55)	0.05 (0.76)	0.9322
Ferritin (ng/mL)	100.63 (366.23)	51.96 (325.06)	0.7362
Bicarbonate (mmol/L)	−1.1 (3.68)	0.57 (3.83)	0.1049
Triglycerides (mg/dL)	6.88 (55.32)	−6.54 (81.95)	0.853
Cholesterol LDL (mg/dL)	4.28 (16.59)	−0.49 (22.06)	0.6395

## Data Availability

The datasets used and/or analyzed during the current study are available from the corresponding author on reasonable request. The data are not publicly available due to privacy.

## Supplementary Materials

The following supporting information can be downloaded at: https://www.mdpi.com/article/10.3390/nu16121968/s1, Table S1: Variable analysis using multiple linear regression.
