# Supplementary material for: The Effect of Intradialytic Exercise Using Virtual Reality on the Body Composition of Patients with Chronic Kidney Disease
Source: Nutrients. 2024 Jun 20;16(12):1968. doi: 10.3390/nu16121968 (PMC11206238; doi:10.3390/nu16121968)
Supplement: Supplementary file 1 [file nutrients-16-01968-s001.zip › nutrients-3035770-supplementary.pdf]

Table S1 Variable analysis using multiple linear regression

Fat tissue index (kg/m²)					
	**Estimate**	**Std. Error**	**Lower 95%**	**Upper 95%**	***p value***
Intercept	1,473	1,284	-1,108	4,054	0,257
Age (Years)	-0,02	0,019	-0,058	0,017	0,282
Gender (Women)	1,404	0,597	0,203	2,605	0,023*
Exercise group	-1,2	0,598	-2,402	0,002	0,05
R Squared	0,2269				
Adj. R Squared	0,1786				

Lean tissue index (kg/m²)					
	**Estimate**	**Std. Error**	**Lower 95%**	**Upper 95%**	***p value***
Intercept	0,445	1,078	-1,722	2,612	0,682
Age (Years)	-0,01	0,016	-0,042	0,021	0,505
Gender (Women)	0,318	0,502	-0,691	1,326	0,53
Exercise group	0,875	0,502	-0,134	1,885	0,088
R Squared	0,0667				
Adj. R Squared	0,0084				

Lean tissue index (kg/m²) from patients with exercise adherence ≥ 40%					
	**Estimate**	**Std. Error**	**Lower 95%**	**Upper 95%**	***p value***
Intercept	1,096	1,051	-1,039	3,23	0,305
Age (Years)	-0,024	0,015	-0,056	0,007	0,124
Gender (Women)	0,926	0,539	-0,169	2,021	0,095
Exercise group	2,11	0,551	0,993	3,228	0,001*
R Squared	0,3137				
Adj. R Squared	0,2548				

Extracellular/intracellular water					
	**Estimate**	**Std. Error**	**Lower 95%**	**Upper 95%**	***p value***
Intercept	-0,006	0,067	-0,141	0,128	0,924
Age (Years)	0,001	0,001	-0,001	0,003	0,281
Gender (Women)	-0,035	0,031	-0,098	0,028	0,266
Exercise group	-0,084	0,031	-0,0146	-0,021	0,01*
R Squared	0,1504				
Adj. R Squared	0,0973				

Phase angle degree					
	**Estimate**	**Std. Error**	**Lower 95%**	**Upper 95%**	***p value***
Intercept	0,734	0,351	0,028	1,439	0,042
Age (Years)	-0,012	0,005	-0,022	-0,002	0,025
Gender (Women)	-0,194	0,163	-0,523	0,134	0,24
Exercise group	0,087	0,163	-0,242	0,415	0,598
R Squared	0,1168				
Adj. R Squared	0,0616				

* p < 0,05
